# Meta-analysis of associations of vascular endothelial growth factor protein levels and -634G/C polymorphism with systemic lupus erythematosus susceptibility

**DOI:** 10.1186/s12881-019-0783-1

**Published:** 2019-03-22

**Authors:** Wenzhuang Tang, Tianbiao Zhou, Zhiqing Zhong, Hongzhen Zhong

**Affiliations:** 1 Department of Blood Purification, the First Affiliated Hospital of Hainan Medical College, Haikou, China; 2 Department of Nephrology, the Second Affiliated Hospital, Shantou University Medical College, No. 69 Dongsha Road, Shantou, 515041 China

**Keywords:** Systemic lupus erythematosus (SLE), Lupus nephritis (LN), Vascular endothelial growth factor (VEGF), -634G/C, Gene polymorphism, Meta-analysis

## Abstract

**Background:**

The purpose of this study was to detect the effects of vascular endothelial growth factor (VEGF) on systemic lupus erythematosus (SLE) risk.

**Methods:**

Associated studies were extracted from the China Biological Medicine Database (CBM), and PubMed on June 10, 2018, and applicable investigations were pooled and analyzed by meta-analysis using RevMan 5.3.

**Results:**

VEGF levels was associated with SLE risk (mean differences (MD) =196.02, 95% CI: 135.29–256.75, *P* < 0.00001), and VEGF levels was associated with active SLE risk (MD =77.51, 95% CI: 10.98–144.05, *P* = 0.02). We also found that VEGF levels was associated with SLE developing into lupus nephritis (LN) risk (MD =223.16, 95% CI: 144.38–301.93, *P* < 0.00001). However, VEGF -634G/C gene polymorphism (rs2010963) was not associated with SLE risk.

**Conclusions:**

VEGF levels was associated with SLE risk, active SLE risk and SLE developing into LN risk. However, there was no an association between VEGF -634G/C gene polymorphism and SLE risk.

## Background

Vascular endothelial growth factor (VEGF), located on chromosome 6 (6p21.1) and a key regulator of vascular formation, is initially characterized by its actions on the endothelial fenestration and maintains permeability of capillary vessels, inducing vasculogenesis, angiogenesis [1, 2]. VEGF, an essential growth factor, is involved in the glomerular development and the postnatal homeostasis, and it is secreted by podocytes into the primary urine in high amounts, back-filtered across the glomerular capillary wall to act on the endothelial cells [3]. VEGF can repair interstitial tubule compartment in the cyclosporine nephrotoxicity, but the mRNA level of VEGF has been up-regulated in the tubules in hypoxic states [2]. VEGF gene polymorphism can affect the activation of VEGF, and there are some studies find that VEGF gene polymorphism is associated with SLE risk.

Systemic lupus erythematosus (SLE), characterized by the formation of immune complexes with nuclear antigens and the production of antibodies to components of the cell nucleus (antinuclear antibodies or ANAs) [4], is a prototypic and heterogeneous autoimmune disease with a wide clinical expression [5], and presents highly heterogeneous clinical manifestations and multi-systemic involvement [6]. Lupus nephritis (LN) is one of the most frequent and crucial complication of SLE, considered as the major predictor of poor prognosis [7]. Some factors were reported that gene expression, protein expression, and gene polymorphism were associated with the risk of some diseases [8–12]. Furthermore, results from meta-analysis are more robust when compared to individual study. There was no meta-analysis to assess the relationship between VEGF levels / VEGF gene polymorphism and SLE risk, and the association of VEGF with SLE developing into LN. In this study, we widely collected the related research on these relationships and used meta-analysis method to pool the results.

## Methods

### Search strategy

The retrieval strategy of vascular endothelial growth factor, VEGF, systemic lupus erythematosus, SLE, lupus nephritis and LN were entered into China Biological Medicine Database (CBM), and PubMed on June 10, 2018, without language limitations. We also checked the references cited in the recruited articles for additional reports.

## Inclusion and exclusion criteria

### Inclusion criteria

(1) The study should be a case-control study; (2) The outcome should be SLE or LN; (3) There were two groups (case group vs control group). (4) Levels of VEGF should be detected by enzyme-linked immunosorbent assay (ELISA).

### Exclusion criteria

(1) Case reports, editorials and review articles; (2) Articles did not provide the VEGF levels or detail genotype data; (3) Association of VEGF genotype / VEGF level with other diseases which were not related to SLE or LN. (4) non-traditional ELISA method for the determination of VEGF levels, such as sandwich ELISA test.

### Data extraction and synthesis

Data and information from each investigation was extracted independently by at least 2 investigators: the surname of first author, publication year, VEGF protein levels, and the sample number of case group and control group for VEGF genotypes. The frequencies of alleles were counted for the SLE group and the control group. The results were compared and disagreements were resolved by discussion.

The diagnosis of SLE was based on according to ACR (American College of Rheumatology) criteria [13]. Active SLE was defined as follows: disease activity score of SLE was evaluated by the systemic lupus erythematosus disease activity index (SLEDAI) score, and a patient was diagnosed as active if SLEDAI score was higher than or equals to 10 [14].

### Statistical analysis

Available data was analyzed using Cochrane Review Manager Version 5 (Cochrane Library, UK). When the *P* value of heterogeneity test was more than 0.1, the pooled statistic was counted using the fixed effects model, otherwise, a random effects model was conducted. Odds ratios (OR) was used to express the results for dichotomous data and mean differences (MD) was used to express the results for continuous data, and 95% confidence intervals (CI) were also counted. A *p*-value of 5% or lower was considered to be statistically significant, and I^2^ was used to test the heterogeneity among recruited studies.

## Results

### Search results

Fifteen articles [14–28] were related to VEGF levels in SLE vs control in this meta-analysis, including 573 SLE patients and 436 controls (Table 1). Six reports [14, 22, 23, 25, 26, 28] were included for the meta-analysis of Active SLE vs Inactive SLE. Two studies [16, 27] were recruited for the meta-analysis of LN vs SLE without LN. Three reports [29–31] were included for the analysis of the effect of VEGF -634G/C gene polymorphism (rs2010963) on SLE risk, including 523 SLE patients and 550 controls.

**Table 1:** characteristics of the studies for the relationship between VEGF levels (pg/ml) and SLE risk

Author, year	Country	Sex (F/M)		Age (Years)			SLE			Control	
		SLE	Control	SLE	Control	Mean	SD	N	Mean	SD	N
Navarro 2002	Mexico	24/4	19/5	36.6 ± 16.1	29.2 ± 8.5	70.25	168.26	28	23.48	153.7	24
Heshmat 2007	Egypt	24/1	29/1	14.1 ± 2.6	14 ± 2.5	579.5	184.7	25	113.2	30.8	30
Kuryliszyn-Moskal 2007	Poland	44/3	NC	40.8 ± 13.6	NC	225	187.5	44	150	140	30
Tanaseanu 2007	Romania	12/3	NC	35 ± 15	NC	744.2	425.1	15	330.3	84.16	10
Ciprandi 2008	Italy	40/0	33/7	41.95 ± 8.3	43 ± 8.2	662.5	700	40	500	512.5	40
Colombo 2009	Italy	80/0	80/0	42.6 ± 9.1	40.1 ± 9.5	307.9	292.2	80	120.7	118.4	80
Kuryliszyn-Moskal 2009	Poland	76/4	NC	39.5 ± 13.3	NC	355.9	292.2	47	144.6	54.4	33
Edelbauer 2012	Austria	17/6	5/15	15 ± 5	12 ± 3	216	28	14	40	9	20
Robak 2013	Poland	55/5	17/3	39.2 ± 27.5	NC	431.9	311.6	60	202.5	117.6	20
Willis 2014	USA	18/3	27/5	44.6 ± 25.5	NC	453.99	261.19	21	113.58	88.29	32
Zhou 2014	China	50/4	22/6	36.81 ± 12.52	37.82 ± 12.86	91.47	108.67	54	47.29	52.62	28
Bărbulescu 2015	Romania	16/2	16/1	45 ± 10.81	NC	68.99	71.06	18	31.84	11.74	17
Liu 2015	China	59/16	31/9	35.42 ± 11.79	33.62 ± 10.21	327.5	702.55	75	172.9	207.45	40
Ghazali 2017	Malaysia	44/2	26/0	32.39 ± 11.46	33.19 ± 10.30	553.65	398.64	46	343	198.21	26
Merayo-Chalico 2018	Mexico	NC	NC	34.6 ± 4.2	36 ± 4.1	653	275	6	223	116	6

Note: *VEGF* vascular endothelial growth factor, *SLE* systemic lupus erythematosus, *SD* standard deviation, *N* the total number of SLE group or control group, *Control* healthy controls, *F/M* female/male, *NC* not clear

### Association of VEGF with SLE risk

**Fig. 1:**
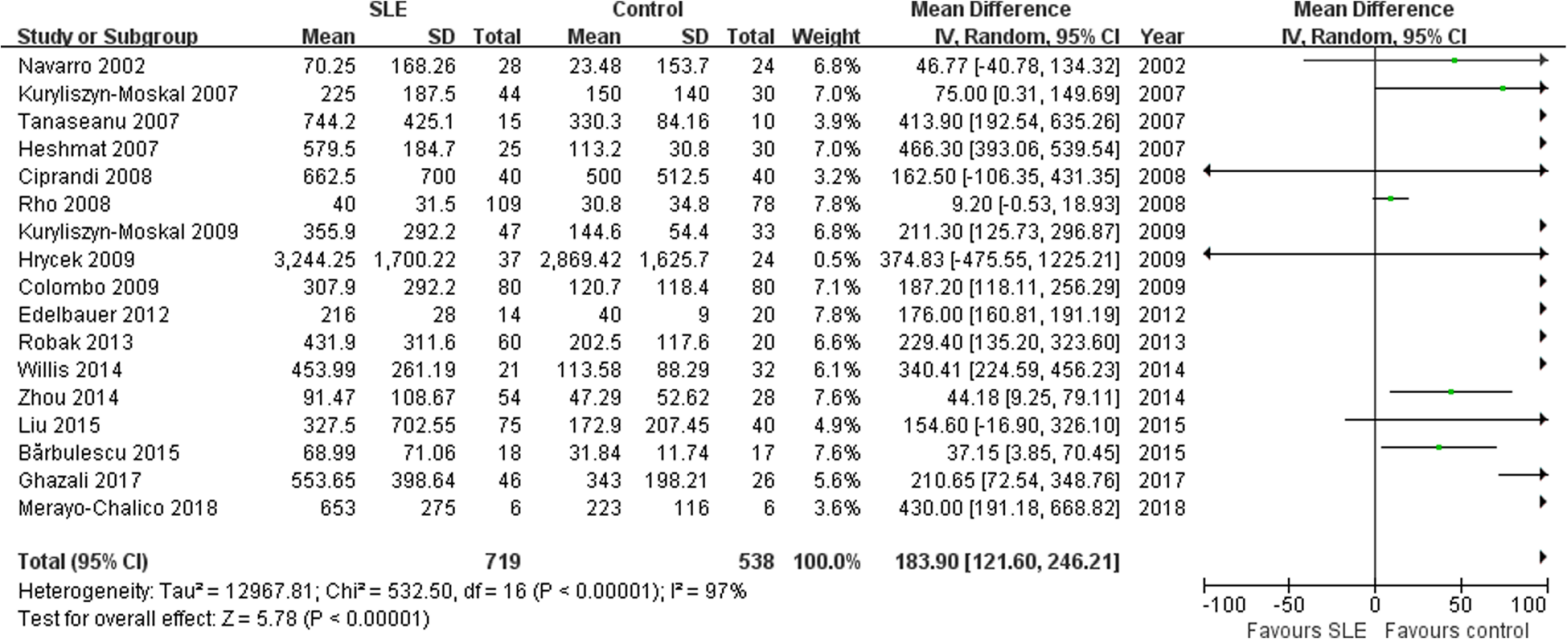
Association of vascular endothelial growth factor protein levels with SLE risk. SLE: systemic lupus erythematosus; SD: standard deviation; Total: the total number of SLE group or control group; CI: confidence intervals; I^2^: test the heterogeneity among recruited studies; df: degrees of freedom

In this meta-analysis, we found that VEGF levels was associated with SLE risk (MD =196.02, 95% CI: 135.29–256.75, *P* < 0.00001; Fig. 1). The *p*-value of the heterogeneity test was < 0.00001. Thus, a random effects model was conducted.

### Association of VEGF with active SLE risk

**Fig. 2:**
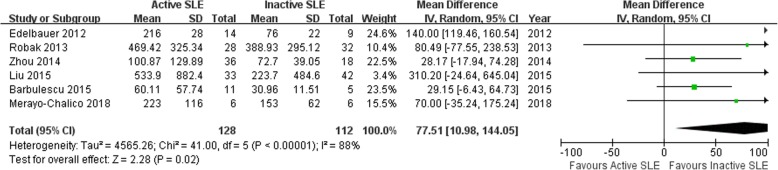
Association of vascular endothelial growth factor protein levels with active SLE risk. SLE: systemic lupus erythematosus; SD: standard deviation; Total: the total number of SLE group or control group; CI: confidence intervals; I^2^: test the heterogeneity among recruited studies; df: degrees of freedom

In this meta-analysis, we found that VEGF levels was associated with active SLE risk (MD =77.51, 95% CI: 10.98–144.05, *P* = 0.02; Fig. 2). The *p*-value of the heterogeneity test was < 0.00001. Thus, a random effects model was conducted.

### Association of VEGF with SLE developing into LN

**Fig. 3:**
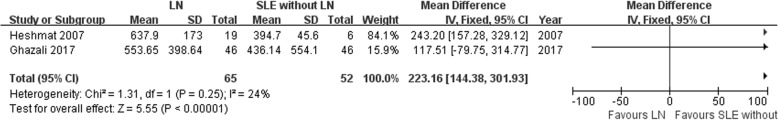
Association of vascular endothelial growth factor protein levels with SLE developing into LN. SLE: systemic lupus erythematosus; LN: Lupus nephritis; SD: standard deviation; Total: the total number of SLE group or control group; CI: confidence intervals; I^2^: test the heterogeneity among recruited studies; df: degrees of freedom

In this meta-analysis, we found that VEGF levels was associated with SLE developing into LN risk (MD =223.16, 95% CI: 144.38–301.93, *P* < 0.00001; Fig. 3). The *p*-value of the heterogeneity test was 0.25. Thus, a fixed effects model was conducted.

### Association of the VEGF -634G/C gene polymorphism with SLE susceptibility

**Fig. 4:**
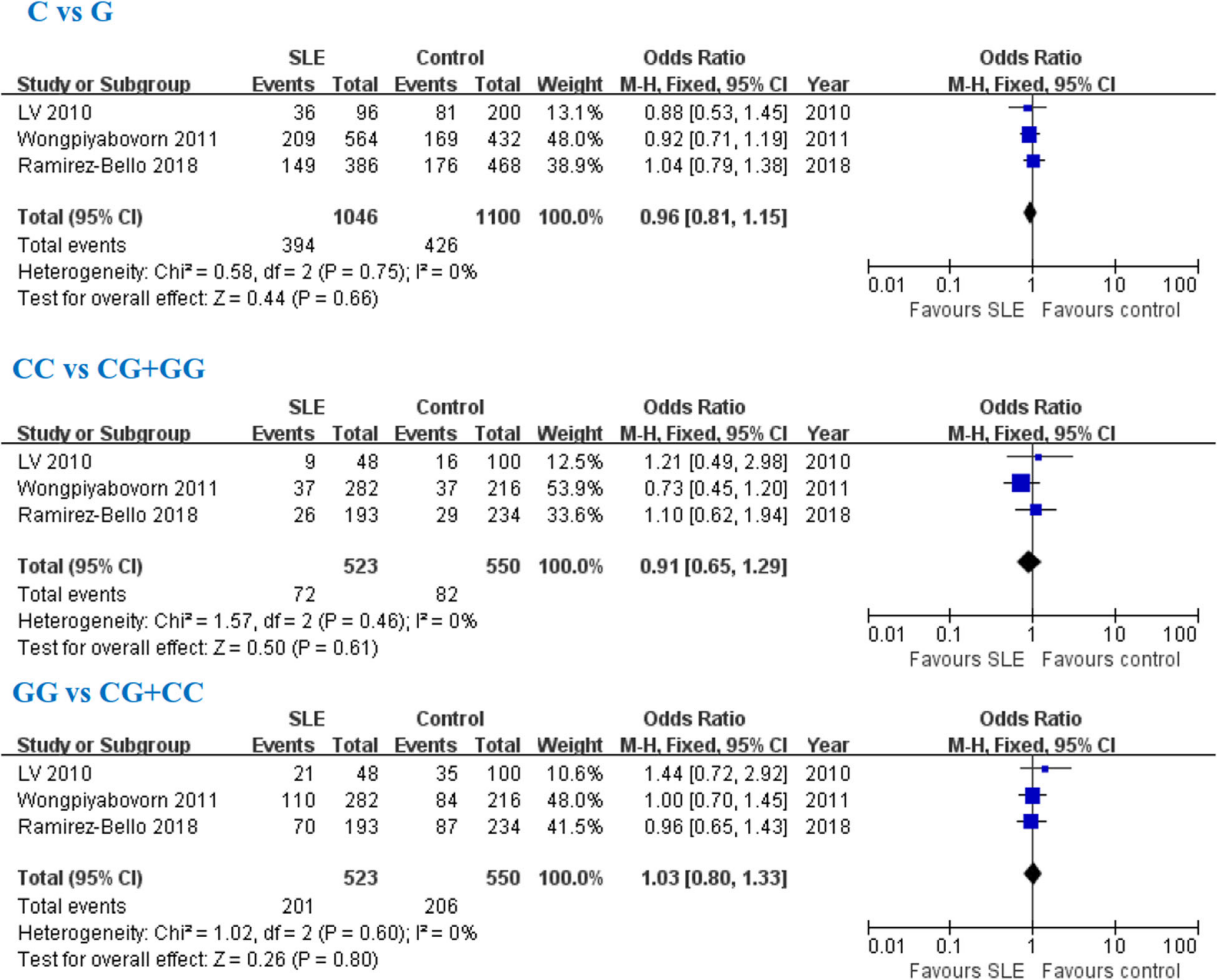
Association between vascular endothelial growth factor -634G/C gene polymorphism (rs2010963) with SLE susceptibility. SLE: systemic lupus erythematosus; M-H: Mantel-Haenszel; Total: the total number of SLE group or control group; CI: confidence intervals; I^2^: test the heterogeneity among recruited studies; df: degrees of freedom

In this meta-analysis, we found that VEGF -634G/C gene polymorphism was not associated with SLE risk (C allele: OR = 0.96, 95% CI: 0.81–1.15, *P* = 0.66; CC genotype: OR = 0.91, 95% CI: 0.65–1.29, *P* = 0.61; GG genotype: OR = 1.03, 95% CI: 0.80–1.33, *P* = 0.80; Fig. 4).

## Discussion

In this study, we found that VEGF levels was associated with SLE risk, and VEGF levels was associated with active SLE risk. We also found that VEGF levels was associated with SLE developing into LN risk. It indicated that VEGF was associated with the SLE risk, the activation of SLE, and the SLE progression. The sample size for the meta-analysis on the relationship between VEGF levels and SLE risk was larger, and the results might be robust. SLE is an autoimmune disease, associated with the primary site represented by vascular endothelial cell injury, and VEGF has been regarded as a key mediator of modulator of neovascularization and endothelial dysfunction. We speculated that the increased VEGF protein levels was associated with the SLE vascular inflammation and associated with SLE risk. However, more studies should be performed to confirm it.

There were some meta-analyses to detect the association between VEGF expression and diseases. Huang et al. [32] included nine articles met the inclusion criteria for our meta-analysis to examine the relationship between the protein expression of VEGF and lymph node metastasis (LNM) in papillary thyroid cancer, and reported that LNM occurred more frequently in papillary thyroid cancer patients with high VEGF expression than in those with low VEGF expression. Fafliora et al. [33] included 11 studies in the meta-analysis, and reported that VEGF levels in patients with malignant pleural effusion were increased as compared to the patients with benign pleural effusion. Lee et al. [34] conducted a meta-analysis of the VEGF levels in patients with rheumatoid arthritis and controls including 13 studies, and found that significantly higher circulating VEGF levels in patients with rheumatoid arthritis and a positive correlation between VEGF levels and disease activity in rheumatoid arthritis. VEGF levels might be a predicted factor for some diseases. In our meta-analysis, we also found that VEGF was associated with SLE risk and SLE progression.

In this study, we also conducted the meta-analysis for the association of VEGF gene polymorphism with SLE susceptibility, and we found that VEGF -634G/C gene polymorphism (rs2010963) was not associated with SLE risk. There was no other meta-analysis to assess this relationship. However, there was only three reports included for the analysis of the effect of VEGF -634G/C gene polymorphism on SLE risk. This result must be treated with caution. More studies should be conducted to confirm the results in the future. We speculated that the VEGF -634G/C gene polymorphism was not associated with the VEGF levels or the activity of VEGF, and it was not associated with SLE risk. However, more investigations should be conducted to confirm it.

In previous, there were some meta-analysis conducted to assess the relationship between VEGF -634G/C gene polymorphism and diseases. Zhuang et al. [35] included nine investigations with 2281 cases with gastric cancer and 2820 controls for meta-analysis, and reported that VEGF -634G/C G allele carrier may increase gastric cancer risk. Gong et al. [36] included six studies in their meta-analysis, and reported that the VEGF -634G/C gene polymorphism was not associated with an increased risk for renal cell carcinoma. Malik et al. [37] included six case-control studies for meta-analysis, and suggested that VEGF-634G/C gene polymorphism might not be associated with retinopathy of prematurity risk. In our meta-analysis, we found that VEGF -634G/C gene polymorphism was not associated with SLE risk.

## Conclusions

In this study, we found that VEGF levels was associated with SLE risk, active SLE risk and SLE developing into LN risk. However, VEGF -634G/C gene polymorphism was not associated with SLE risk.

## Abbreviations

CBM: China Biological Medicine Database
CI: confidence intervals
LN: Lupus nephritis
LNM: lymph node metastasis
MD: mean differences
OR: Odds ratios
SLE: systemic lupus erythematosus
VEGF: vascular endothelial growth factor
